# A Two-Layer IP Hopping-Based Moving Target Defense Approach to Enhancing the Security of Mobile Ad-Hoc Networks

**DOI:** 10.3390/s21072355

**Published:** 2021-03-28

**Authors:** Pengkun Wang, Momiao Zhou, Zhizhong Ding

**Affiliations:** 1 School of Computer and Information, Hefei University of Technology, Hefei 230009, China; wpk2018110948@mail.hfut.edu.cn (P.W.); mmzhou@hfut.edu.cn (M.Z.); 2 Anhui Province Key Laboratory, Industry Safety and Emergency Technology, Hefei University of Technology, Hefei 230009, China

**Keywords:** MANET, network security, IP hopping, moving target defense, anti-intrusion

## Abstract

Mobile ad-hoc networks (MANETs) have great potential applications in military missions or emergency rescue due to their no-infrastructure, self-organizing and multi hop capability characteristics. Obviously, it is important to implement a low-cost and efficient mechanism of anti-invasion, anti-eavesdropping and anti-attack in MANETs, especially for military scenarios. The purpose of intruding or attacking a MANET is usually different from that of wired Internet networks whose security mechanism has been widely explored and implemented. For MANETs, moving target defense (MTD) is a suitable mechanism to enhance the network security, whose basic idea is to continuously and randomly change the system parameters or configuration to create inaccessibility for intruders and attackers. In this paper, a two-layer IP hopping-based MTD approach is proposed, in which device IP addresses or virtual IP addresses change or hop according to the network security status and requirements. The proposed MTD scheme based on the two-layer IP hopping has two major advantages in terms of network security. First, the device IP address of each device is not exposed to the wireless physical channel at all. Second, the two-layer IP hops with individual interval and rules to obtain enhanced security of MANET while maintaining relatively low computational load and communication cost for network control and synchronization. The proposed MTD scheme is implemented in our developed MANET terminals, providing three level of network security: anti-intrusion in normal environment, intrusion detection in offensive environment and anti-eavesdropping in a hostile environment by combining the data encryption technology.

## 1. Introduction

Mobile ad-hoc networks (MANETs) are playing an increasingly important role in many environments and applications, for example, in emergency environments where fixed network infrastructure might be damaged. MANETs used in military applications might face a hostile environment, which means that the network might be intruded and eavesdropped.

Network intrusion and eavesdrop attacks in a wired network can be primarily divided into passive and active ones based on their characteristics. Passive attacks are designed to rebuild the network topology or to analyze traffic and mobility patterns, while active attacks change data by inserting wrong data packets or by modifying the contents of data packets. The traditional defense method is usually by the use of identity verification and the trusted certificate authority authorization. For MANET, however, authentication and authorization are not suitable if the feature of no-infrastructure or no central node should be kept.

Mobile Target Defense (MTD) [1,2] is an innovative defense mechanism that can change the network defense mode or network configuration irregularly with the passage of time. The IP hopping of MTD is a typical MTD mechanism, which prevents attackers from eavesdropping and intrusion by dynamically changing IP addresses [3]. The use of IP hopping technology can not only prevent illegal users from intruding into the network to identify the commander, for example, but also prevent them from grabbing the operational intention and situation by analyzing the network and communication procedure.

To enhance the network security of our developed MANET communication terminals [4,5] in its upgrade version, a two-layer IP hopping-based MTD approach is designed and implemented on an Android platform. The low layer IP that will be transmitted in wireless physical channel is virtual IP address that is generated from the device IP address. The device IP, i.e. the upper layer IP, hops controlled by its own controller. The network is virtually connected with the technology similar to a virtual private network (VPN). The upper layer IP hopping is controlled by its own algorithm. Combined with the technology of data encryption, the implemented MANET system has three level of network security: anti-intrusion in normal environment, intrusion detection in offensive environment and anti-eavesdropping in a hostile environment.

The rest of the paper is organized as follows: The research works related to ours are explored in Section 2. In Section 3, the mechanism, model and algorithm of our approach are introduced in detail. The tests concerning effectiveness and the performance evaluation are presented in Section 4 and conclusions are outlined in the final section.

## 2. Related Work

MTD prevents network attacks by dynamically changing the attack surface of the system such as the static configuration of the network, thereby invalidating the intelligence collected by the attacker and depleting their device resources. According to its mechanism of defense, MTD can be divided into three categories [6]: shuffling-based MTD [7,8,9], diversity-based MTD [10,11,12] and redundancy-based MTD [13,14]. The shuffling- based MTD on is the most common one, which protects networks from attack by rearranging or randomizing the key parameters or information of the system, for example, IP shuffling, port hopping, or randomizing packet headers. Diversity-based MTD employs different implementations of the same functionality or service, and it introduces also in some cases the diversity of software stacks to enhance network resilience or the diversity of programming languages to avoid code injection attacks. The redundancy-based MTD improves system reliability by creating multiple copies of network components, for example, backups of network sessions in a cyber-physical system.

Most MTD anti-intrusion and anti-intrusion technologies based on reorganization mainly focus on IP reorganization. According to the different ways of IP reorganization, it can be divided into three methods: hidden device IP [15,16], device IP redistribution [17,18,19], virtual IP and device IP hopping [20,21,22,23]. To hide the device IP is to pack or modify the device IP in various ways. The document [16] proposed a dynamic defense mechanism based on IPv6, IPv6 network to allow nodes to bind new IPv6 addresses seamlessly. The tunnel technology encapsulates the original data packet, and the source IP address and destination IP address of the tunnel will be changed, making it difficult for an attacker to track the network eavesdropping communication traffic. However, because of its lack of active configuration support for hopping time, it is difficult to apply to MANETs. Device IP redistribution refers to the distribution and networking of a unified new IP to all legal nodes through the server after a period of time. The article [17] used an improved dynamic host configuration protocol server to reassign the host’s IP address, and uses domain name system to locate the current IP based on the host name. The algorithm can protect the IP list from worm attacks, and effectively defend the IP address-based worm propagation attack list. However, because NASR uses local area network addresses, the range of address hopping is limited and not applicable to MANETs. Virtual IP and device IP hopping means that legitimate nodes use virtual identities to communicate and periodically change their virtual identities. They are mainly divided into two types: centralized and distributed control IP hopping. The centralized type, such as described in [21], proposed a way to use a software defined network. Device IP hopping and distribution are implemented on the data plane and switches, and one-way hash chains and data communication protocols are used to synchronize device IP. Since a new IP is generated on the data plane, the overhead is small. However, MANETs without an infrastructure and no central node cannot build controllers and switches for IP distribution and generation. The distributed type, such as the distributed method in essay [23]. Each node established its own IP pool and provided a mechanism to convert virtual IP and device IP to each other. Not only can it withstand many types of active attacks, but it can also reduce the overhead required for IP synchronization by modifying the IP update frequency. However, the network layer protocol needs to be modified, which is not suitable for the rapid combat characteristics of military MANETs.

The abovementioned characteristics of IP hopping security mechanisms are listed in Table 1. Most of the approaches are not suitable for MANETs since they need a central network node to provide centralized service, such as DHCP, user authentication, IP hopping controlling, etc., which is contradictory to non-infrastructure of MANETs. Secondly, the actual device IP addresses in the approaches are exposed to wireless physical channel, which is a risk of being intruding. Thirdly, they adopt only one layer IP hopping, device or virtual. Fourthly, the trigger of IP hopping in them is either by time or by event, which is not flexible. In order to overcome those shortcomings, we propose an approach in this paper, whose features are list in the last column of Table 1. The proposed approach has been implemented in our developed MANET terminals.

## 3. Two-Layer IP Hopping Approach

**Figure 1:**
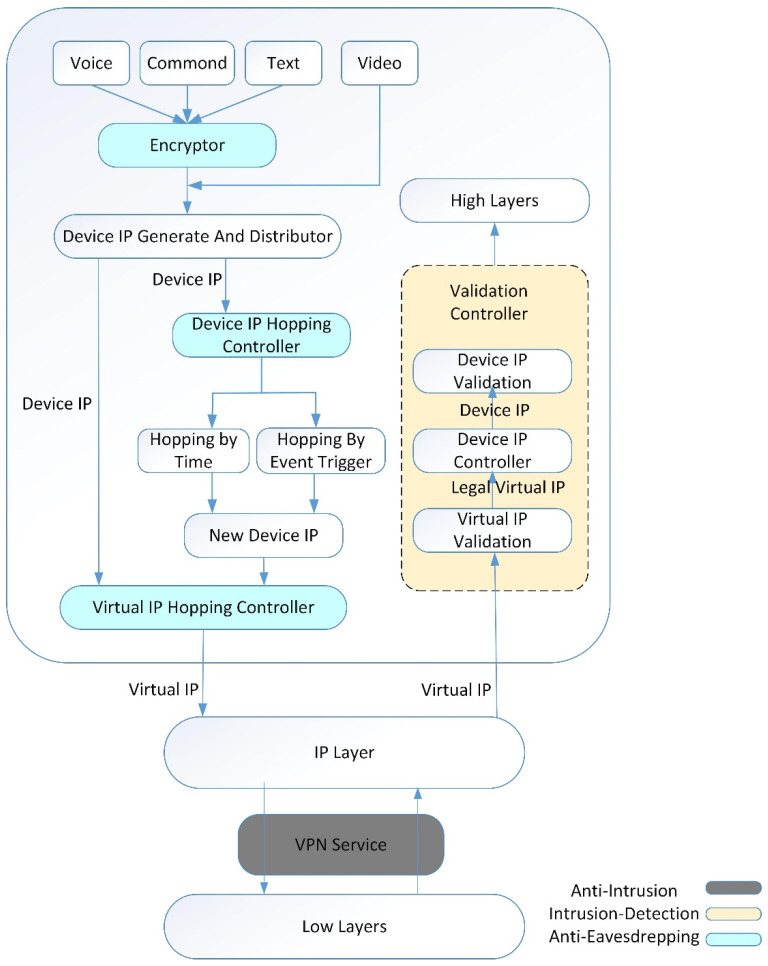
Proposed security scheme embedded two-layer IP hopping.

In a wireless network, all the data flow including network parameters will be exposed to all receivers no matter whether they are legal or illegal users, and the destination IP address has to be transmitted in plaintext in order to realize a point-to-point transmission. This results in the possibility that hostile devices can intrude into the network via the eavesdropped IP address segment, for example, in a military application. On the other hand, MANETs are a type of no-central node and self-organizing network. Normally they also have no firewall or authentication system due to their limited resources. In order to support the virtues of self-organizing and no-central-node of MANETs, a feasible and efficient defense solution for MANETs is hiding, duping, or dynamically changing their network parameters, especially the IP addresses. The designed and implemented two-layer IP hopping approach is shown in Figure 1, based on which a three level of network security system is constructed.

The three levels of network security are as follows: The use of VPN technology, that is, the VPN service interface under Android, for data transmission not only enhances the security of virtual IP address transmission, but also the device IP address of each device is not exposed to the wireless physical channel at all, so that anti-intrusion occurs in a normal environment. Secondly, the data packet verifies the validity of the virtual IP and the device IP of the node by validation controller. The virtual IP in the data packet detects its legitimacy through the virtual IP validation, and then the legal virtual IP is converted into a device IP. The device IP is compared with the routing table to check its legitimacy, so that intrusion detection works in an offensive environment. Finally, the virtual IP hopping mechanism before each data packet transmission and the device IP hopping mechanism combine the on-time hopping and the event-triggered hopping during the data transmission process to not only enhance the security of the MANET, but also enable network control and synchronization. The calculation volume and communication cost of the system are kept low, thus realizing anti-eavesdropping in a hostile environment. Our solution is very practical, because it can construct and encode the device IP addresses, and randomly assign and construct a modular solution, without considering the implementation of routing rules, and it will not incur in any other overhead except for the overhead of the seed distribution network.

### 3.1. Encryption Algorithm

In order to prevent the data in the packet from being cracked, the AES algorithm is chosen for encryption because it is faster and more secure than other encryption algorithms [24,25,26]. AES is a typical symmetric encryption algorithm for symmetric block encryption [27]. It is noticed that there are some AES-related encryption algorithms proposed recently, for example [28,29], which provides better performance for encrypting and transferring image data than for text data. In contrary, AES is more efficient for text encryption, which is our case.

**Figure 2:**
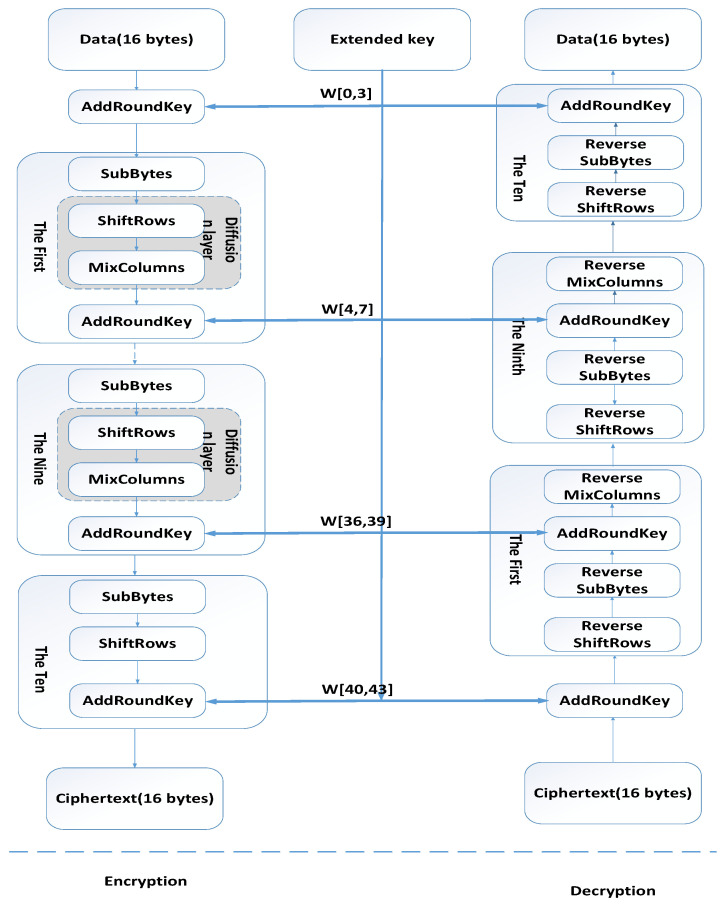
AES algorithm.

As shown in Figure 2, when encrypting data, each round of AES encryption cycle except the last round includes four steps: AddRoundKey, SubBytes, ShiftRows and MixColumns. With AddRoundKey, in each encryption cycle, the master key will generate a round key, the key size will be the same as the original matrix, and each corresponding byte in the original matrix will be XORed (⊕) Add. With SubBytes, bytes are replaced by replacement boxes. With ShiftRows, bytes are shifted in a row of the array state and the offset is different in each row. With MixColumns, data is merged in each column of the array status.

### 3.2. Access Randomization Scheme: Virtual IP Hopping

Considering the multi-hop situation of MANET, we perform virtual IP hopping on all nodes involved in each path. We use device IP ($IP_{seed}$) and Mersenne twister seed ($MT_{seed}$) to generate a virtual IP address ($IP_{update}$), and use VPN technology to replace the device IP with a virtual IP before sending the data packet. In addition, considering how other nodes can judge the legitimacy of the hopping node after receiving the data packet after the virtual IP jumps, we provide a method for judging whether the node is legal after receiving the data packet, that is, the conversion between the device IP address and the virtual IP address. The process of converting the device IP address to the virtual IP address is called IP hopping, and the reverse process is called IP de-hopping.

**Figure 3:**
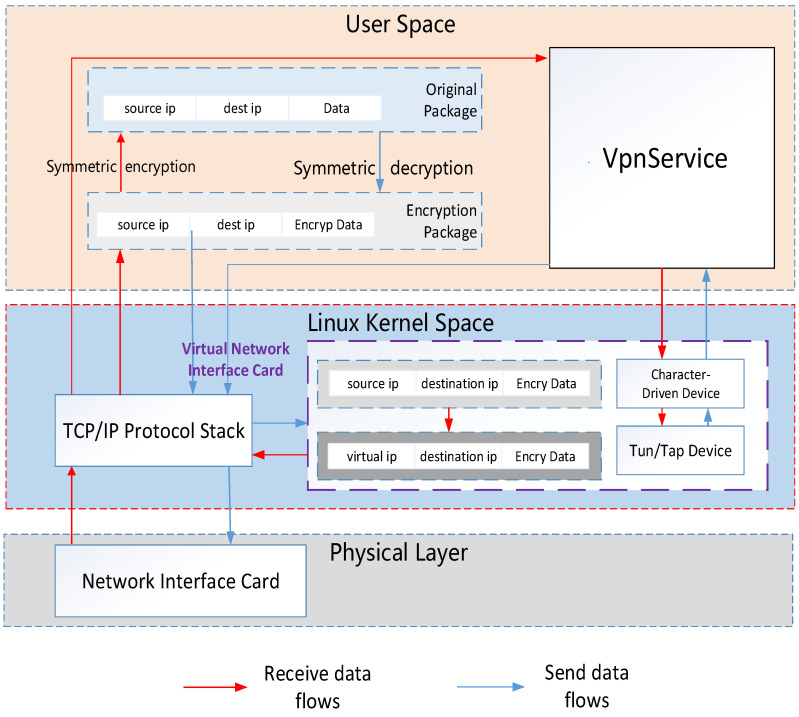
The overall data transfer framework.

**Figure 4:**
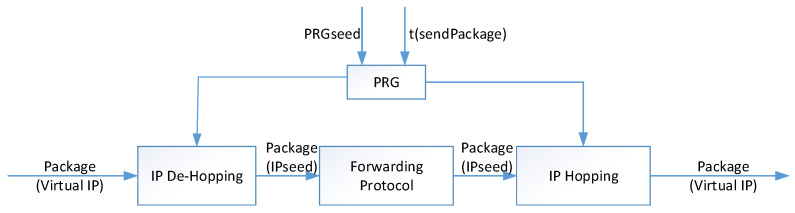
Generation of virtual IP address.

As shown in Figure 3, before each program starts running, the algorithm assigns a unique device IP and Mersenne twister in the control room. All nodes know each other’s device IP addresses and establish their own independent routing table to identify different devices. Then, each device starts to generate its own virtual IP for communication. The device generates update IP address ($IP_{update}$) through two seeds: a unique static IP seed ($IP_{seed}$) for each device and a random Mersenne Twister seed ($MT_{seed}$). $IP_{update}$ is a function f of the $IP_{seed}$ and Mersenne twister output, which in turn $IP_{update}$ is a new $MT_{seed}$:

(1) $$IP_{update}=f(IP_{seed},MT(t,MT_{seed}))$$

where function f are deterministic functions. This IP address translation is also described in Figure 4. The function *f* is easy to calculate, while the Mersenne twister is hard to calculate.

A series of word vectors are generated by the Mersenne twister, and these word vectors are treated as uniform pseudo-random numbers between 0 and $2^{w}$—1. Dividing by $2^{w}$—1, each word vector is generated in the real number [0, 1]. A word x the recurrence relation as follows:

(2) $$x_{k+n}=x_{k+m}⊕(x_{k}^{u}|x_{k+l}^{l})A,\left(k=0,1\ldots \right)$$

*X* applies the top mask and bottom mask respectively. Select the format of matrix *A* to make *A* is multiplication very fast. The equation of *A* matrix is as follows:

(3) $$A=R=\left(\begin{array}{cc} 0 & I_{w-1}\\ a_{w-1} & \left(a_{w-2,\ldots }a_{0}\right) \end{array}\right)$$

As a (*n* − 1) × (*n* − 1) identity matrix, unlike normal matrix multiplication, bitwise XOR is used instead of addition. The advantage of the rational paradigm is that it can be effectively expressed as:

(4) $$a=(a_{w-1},a_{w-2},\ldots ,a_{0})$$

(5) $$x=(x_{w-1},x_{w-2},\ldots ,x_{0})$$

where *x* is:

(6) $$x=(x_{k}^{u}{|x}_{k+1}^{l}),\left(k=0,1\ldots \right)$$

where *a* is:

(7) $$a=(a_{w-1},a_{w-2},\ldots ,a_{0}),x=(x_{w-1},x_{w-2},\ldots ,x_{0})$$

The Mersenne twister can also be written as:

(8) $$x_{k+n}=x_{k+m}+x_{k+1}\left(\begin{array}{cc} 0 & 0\\ 0 & I_{r} \end{array}\right)A+x_{k}\left(\begin{array}{cc} I_{w-r} & 0\\ 0 & 0 \end{array}\right)A$$

In our implementation, we realize IP hopping by applying the following points: randomization is at the device IP, for example, we used Mersenne twister based on a linear feedback shift register (t = 0 corresponds to no shift and is in the state of $MT_{seed}$), function *f* is the cyclic addition of each decimal. Therefore, in our implementation, f and Mersenne twister are linear and computationally efficient. $IP_{seed}$ can be found by reversing the operation and using the *f*^−1^ in the $IP_{update}$, or circular subtraction of the Mersenne twister output, as shown in Figure 2. For time *t* packets, the legitimate user of the forwarding packet is aware of the Mersenne twister output because they use the same Mersenne twister, $MT_{seed}$, *f*, and *t*. After the network finishes sending the data packet, the forwarding node first takes out the source IP ($IP_{update}$) in the data packet. Firstly, $MT_{seed}$ is calculated by the time t in the data packet, and then $MT_{seed}$ and $IP_{update}$ are used to perform circular subtraction to calculate $IP_{seed}$, and the routing table is searched through $IP_{seed}$. If the IP address is valid and there is a corresponding route in the routing rules, the next node will be found according to the route.

### 3.3. Virtual IP Hopping Randomization Analyses

In this section, we consider the case of multiple routes. If there are multiple routes at the same time, and the paths carry data packets from multiple source nodes, there may be conflicts in Figure 1. However, if each hop path uses a different Mersenne twister, that is, using different Mersenne twisters will produce different $MT_{seed}$, then we can resolve conflicts and distinguish between multiple paths. In other words, if two data packets from different paths arrive at the Android phone with the same IP address, the two data packets can be distinguished by calculating and identifying the $MT_{seed}$ of each path. The security of this algorithm depends on the confidentiality of $IP_{update}$, which in turn depends on the confidentiality of MT output.

In order to prevent an attacker from pretending to be a legitimate device to enter the network and send data packets, the IP update speed is faster than the attacker’s response time. On the one hand, our IP generation and update are performed inside the node. On the other hand, the virtual IP to be used next time has been calculated before sending data each time, so our solution is very fast. In addition, our solution uses a different time t to ensure that the virtual IP generated by each data packet is different, which prevents attackers from monitoring traffic for a long time and using IP address collection history to obtain information.

### 3.4. Access Randomization Scheme: Device IP Hopping

Device IP hopping algorithm uses a large number of identity pools to protect the device IP of the node. Each node can have multiple device IP pools to perform the device IP hopping, and only legitimate nodes can associate an IP pool with a node’s device IP. IP pools can be preloaded on a node or calculated at run time. In this article, we will use the hash chain to generate the IP pool of each node during the operation of the node. The introduction of effective IP hopping time prevents IP attackers from collecting IP over a period of time and using IP addresses to pretend to be legitimate users to enter the network to send and receive data packets of that IP. Each node uses the IP in the IP pool for a valid period of time. After one IP pool is used up, a new IP pool needs to be regenerated. In order to protect the legitimate nodes on the network to send data packets safely, we propose a mechanism whereby the legitimate nodes can identify the IPs of other legitimate nodes in the network. After the node receives the data packet, it first detects whether the node has an IP hopping through the hash chain, and if it does, it updates its routing table. If there is no IP hopping and the IP does not exist in the routing table, there is an intrusion node. Once an intrusion is detected, the event trigger mechanism of the current node will be triggered to perform the current node device IP hopping.

Information about the state of the network and currently valid IPs, are stored in the transition table by each node and updated regularly through the update mechanism designed to provide node identity authentication and data integrity. To prevent an attacker from modifying or spoofing data, when nodes receive a packet from another device, they compare the packet’s destination IP address to determine whether the IP addresses in the table are the same. If a match is found, the route is determined based on the local route table and the packet is sending to the next hop for that route. If the current IP address jumps, you only need to change the device IP in the originally sent data packet to the new device IP. Therefore, compared with other algorithms that require additional synchronization data packets, our algorithm has no additional communication overhead.

Hash chain was the first proposed password protection scheme for anti-intrusion and anti-eavesdropping attacks [30], and because of the low computational cost of the hash chain, it is widely used in one-time cryptographic signature programs. In this article, we use hash chains to generate IP pools. We assume two properties of the hashing function h which is typical in many encryption applications, the hash function generates pseudo-random numbers and the function has a one-way irreversible characteristic. Providing an input hash function is easy to calculate the output, but providing an output is difficult to calculate the input value. When the IP pool in the one-way hash function h is exhausted or an intrusion occurs, the new hash function is updated through the update of the shared key s.

**Figure 5:**
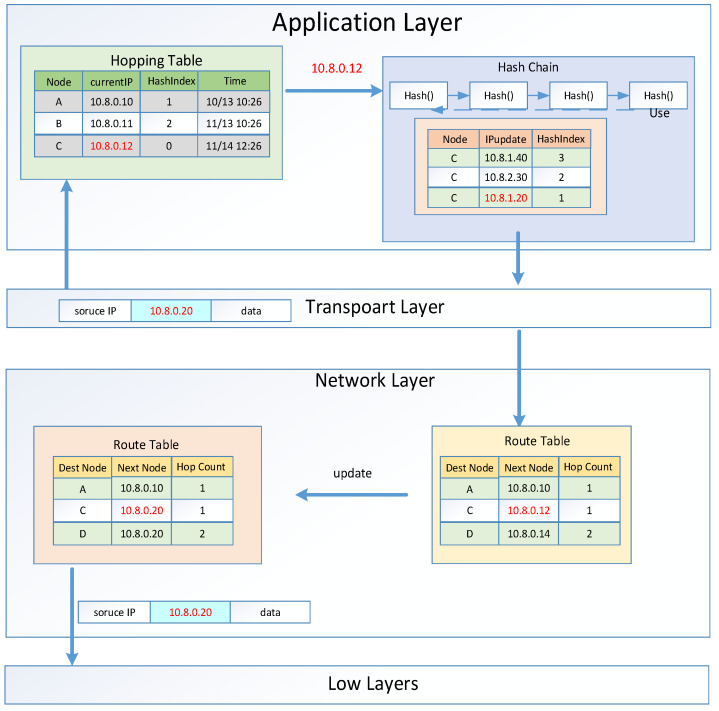
Device IP address hop diagram.

As shown in Figure 5, each node estimates the next hop address of other nodes on the same path in advance by calculating the hash chain of each path. The use of the hash chain is opposite to the direction of generation. The IP hopping are calculated in the forward direction and then these IPs are used in the reverse direction. The construction and use principle based on IP pool is similar to one-time password and token generation [31,32] and wireless network-based broadcasting and authentication [33,34]. Specifically, the conversion method of the hash function is as follows:

(9) $$h^{n}=h^{n-1}(s,h^{n-2})$$

In order to prevent illegal nodes from entering the network, all nodes joining the network first send network access requests to the authenticated nodes in the network, and the nodes join the network or leave the network to request identity verification. As a MANET has no central node and no infrastructure, it is difficult to verify the identity of the node. In this section, we assume that two shared secret keys are provided for all valid nodes in the network: the key k used to encrypt the data in the data packet and the key used by each node to modify the hash parameters after the hash chain is used up. Considering that there may be the same IP after the node jumps, once the node generates a new IP address after the jump, it first broadcasts its own IP address, and other nodes start to compare it with their own IP address after receiving it, if different data packets are directly discarded, if the same, the same data packet is broadcast to indicate that the current IP is unavailable.

Once the timer in the routing table of each node reaches the predetermined time, the node can update the IP address autonomously. This update does not need to exchange synchronization information or control information in the network, but because it is a time-based jump, it needs to rely on a strict time synchronization mechanism. If a network is composed of hundreds of nodes, the IP hopping time is too short, which may cause the previous data packet to not be sent to the target node, causing important information may be lost. If the IP hopping time is too long, the effectiveness of IP hopping will be reduced and the overhead of IP hopping will increase.

If a distributed method is used to update the IP address of each node using a combination of hopping by time and hopping by event-trigger, the ability to prevent intrusion and eavesdropping can be maximized. Therefore, we assume that each node will IP hopping between the minimum hop interval $T_{min}$ and the maximum hop interval $T_{min}$. In the interval [$T_{min}$, $T_{min}$), maximum time is maximum IP validity interval effectiveness, the interval time is greater than the highest disable MTD mechanism, and the smallest $T_{min}$ IP validity interval allows effectiveness (that is, the interval is less than the $T_{min}$ not give enough time to update the information transmission through the network at the next update trigger). $IP_{i}$(k) in just as effective interval delta Δ$T_{i}\left(k\right)$ is used by the node I. When related to the validity of the interval timer expires, Node I will replace its current $IP_{i}$(k) with the next one in the IP chain $IP_{i}$(k−1). Network-wide synchronization interval $T_{sym}$, device IP hopping time interval $T_{hop}$, the number of hopping between two full network synchronizations (the number of nested hash functions) $N_{hop}$, The relationship is as follows:

(10) $$T_{\mathrm{sym}}=T_{hop}*N_{hop}$$

## 4. Tests and Performance Evaluation

In order to evaluate our solution, we simulated a military battle in Section 4.1 and built a MANET environment based on the Android platform. In a single-stream environment with no other network traffic, first all users of the operation are assigned their static IP addresses through the server, and then the initial Mersenne twister seed, hash function, and hash seed are distributed. Section 4.2 measures packet delay and packet loss rate. Section 4.3 discusses the IP hopping mechanism prevents network scanning from intrusion. In Section 4.4, the delay overhead of synchronizing the new hash chain after IP hopping. Finally, in Section 4.5, we compare the three-tier intrusion prevention scheme with the existing intrusion prevention scheme.

### 4.1. Prototype Implementation Based on Android

**Figure 6:**
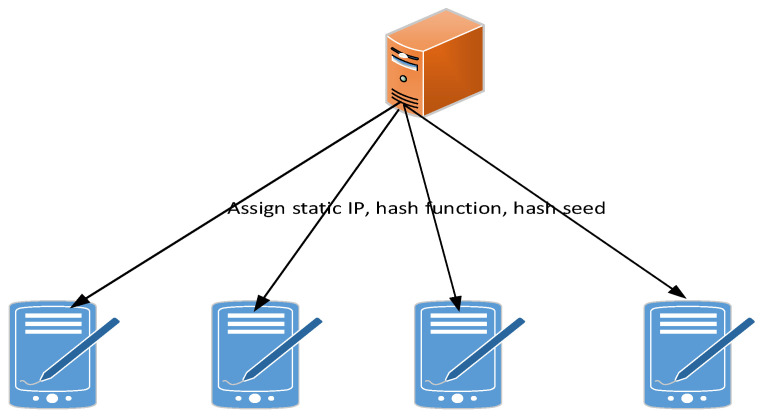
Initialization configuration.

**Figure 7:**
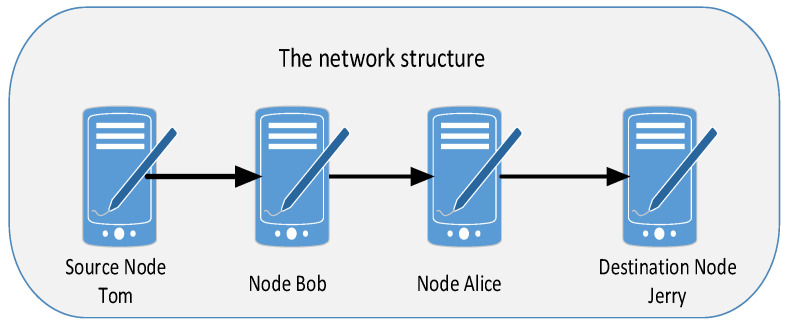
Network Structure.

In order to build a small scale of MANET platform for implementing and testing the proposed approach, four nodes of a MANET is built up by our developed MANET terminals (Exynos4412, 2 GB RAM,16 GB storage, Android 6.0). A source node Tom and a target node Jerry are assumed. Figure 6 and Figure 7 depict the roles of each node in the network topology. We focused on evaluating our solution given a forwarding path.

We implemented the prototype for our solution. In order to ensure the security of data transmission, soldiers are assigned to the static IP, hash function and hash parameters of each host at one time in the secure server through the structure in Figure 6 and UDP protocol before battle. The node then randomizes its IP address locally and sends a packet with an updated IP address. Packets can only pass if the IP address is correct. For example, when node Tom directs its packets to other hosts with incorrect IP addresses such as due to unauthorized and incorrect IP updates, the packets are lost at the next hop of node Jerry.

### 4.2. Random Hopping between Virtual IP and Device IP

For virtual IP hopping we use a Mersenne twister. A Mersenne twister (MT) is a classical method of generating pseudo-random numbers. It is the most widely used method of generating random numbers and is integrated as the default pseudo-random number generator (PRNG) into many software systems, such as Microsoft Visual C++, Python, etc. IP-hopping uses the SHA-256 hash function [35], which has been widely used in security applications due to its mature unidirectional nature. Our scheme relies on the one-way properties of hash functions to prevent an attacker from breaking synchronization by knowing future IP before using them. SHA-256 used in the currency of mining is based on the inverse hash function, search and miners have computing resources globally successful mining, accept multiple solution/collision every 10 min, with such a computing resource is very difficult and expensive, but even assuming that the attacker’s computing resources, breaking the hash chain design synchronous than mining more difficult, because we don’t allow conflict synchronous n.

**Figure 8:**
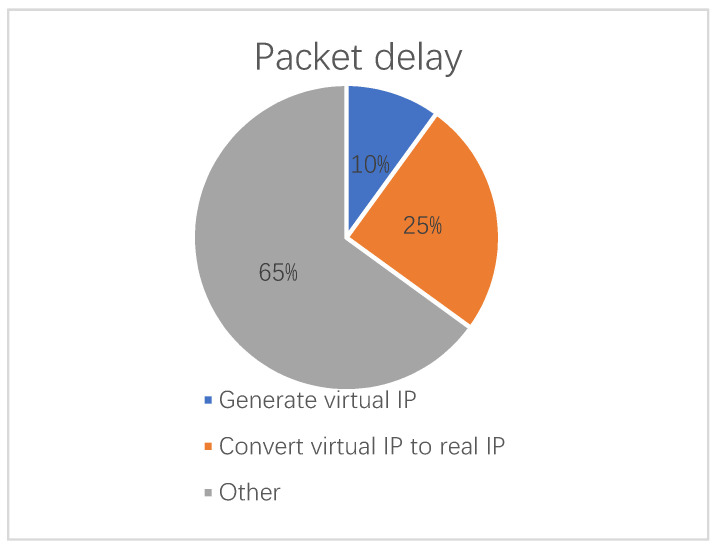
Package delay.

We used Wireshark to monitor network packets for analysis by opening hotspots on the PC side and connecting hotspots through mobile phones and proved that the source IP address in each data transmission packet was a virtual IP. Our solution is different from using a controller to uniformly assign IPs, because we randomly assign the addresses of all nodes on the forwarding path, not just the destination node. The gain of our scheme is obvious because the randomization is done locally inside the node rather than involving the controller. After distributing the Mersenne twister seed to each node, we generate packets and randomize the source IP address of each packet. We also measure latency when randomization is done locally by Mersenne Twister. Figure 8 shows for VPN-based virtual IP packets, each packet had a 2.6586 millisecond delay averaging over 10,000 measurements and a Mersenne Twister for packet transmission of 0.0264 milliseconds, accounting for 0.993% of the total packet delay.

### 4.3. IP Hopping against Eavesdropping

**Figure 9:**
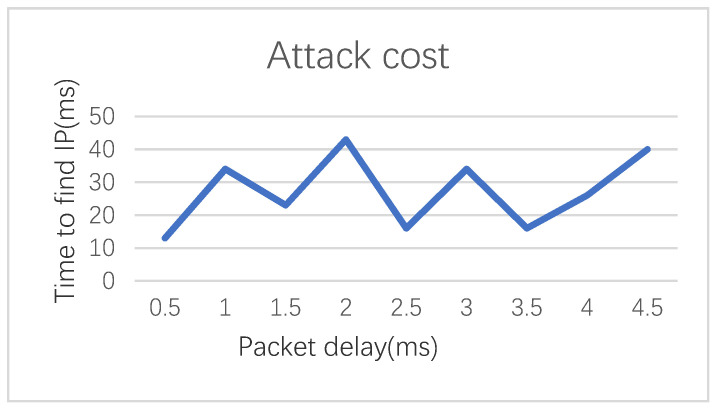
The overhead of realizing network reconnaissance by an attacker.

In order to ensure the unique IP address, VPN technology is used to expand the available range of IP addresses, and broadcast after generating a virtual IP to determine whether the current IP has been used. First, a virtual IP address is randomly generated according to the algorithm, and then the IP address is broadcast to ensure that the IP address is not used. If there is no reply to a data packet with the same IP address within a certain period of time, it means that the IP address is available. In order to test the anti-eavesdropping and anti-intrusion capabilities of the algorithm, the IP address of the node is queried by tracking the route of the data packet, and the relationship between the attacker’s attack cost and the IP jump is calculated in our laboratory. Figure 9 shows that our algorithm can prevent such attacks.

Compared with the average time of 20.56 milliseconds for the attacker to wait for an attack, our algorithm only needs an IP hopping time of 26.4 nanoseconds. Therefore, our algorithm, the attack can only be successful if the reconnaissance delay is more than seven times greater than the packet delivery delay. In other words, if the attacker invades the network by investigating the data packet for too long, then the IP has hopped and the data packet is invalid.

### 4.4. The Cost of Synchronization after IP Hopping

We calculated the cost of the IP synchronization solution. We calculate the time required to successfully transmit data packets again after each node jumps. Due to the one-way irreversibility of the hash function, the SHA-256 hash function is used. As shown in Table 2, JAVA needs 9.5 μs to calculate the SHA-256 function, while the total cost of hash calculation and IP address field update is 10.5 μs. When all the IP addresses in the hash pool are used up or the node detects an intrusion, all parameters need to be changed. At this time, a random function will be used to generate the 0.1 μs required for the new hash parameter, and then the new hash parameter will follow the packets are broadcast to the network together. Since the remaining hash chains become shorter and shorter with the use of IP addresses, and a new chain is urgently needed, the calculation of signature generation synchronization can also be performed offline or in advance.

### 4.5. A Comparison of Intrusion Prevention Scheme

We compared our method with the existing IP hopping method. As shown in Table 3, the results show that it is very simple to deploy this method in real life and it does not require other terminal operating systems or deployed hardware devices. In addition, due to the use of VPN technology, in the process of dynamic address changes, the range of host IP address changes is no longer restricted. Finally, because the device IP and virtual IP are hopped and synchronized within each node in a distributed manner, even if the location of the network node changes multiple times due to multiple movements of MANET, data packets can still be transmitted stably.

## 5. Conclusions

This paper proposes a two-layer IP hopping-based MTD approach to enhance the security of our developed MANET terminal device. In the proposed approach, the device IP address is not exposed to the wireless physical channel at all, and the virtual IP and device IP are triggered to hop either by time or by event. By combining with data encryption technology, the implemented MANET terminal has three levels of network security: anti-intrusion in a normal environment, intrusion detection in an offensive environment and anti-eavesdropping in a hostile environment, while maintaining relatively low computational load and communication cost for network control and synchronization. Our experiments have shown that it is difficult for an attacker to send packets disguised as a legitimate node during the effective time of an IP hopping because it takes at least seven times as long as our hop time to scan for our legitimate IP.

## Figures and Tables

**Table 1:** Comparison of IP hopping schemes.

	References	[15]	[16]	[17]	[18]	[19]	[20]	[21]	[22]	[23]	Ours
Topology	Needs central node	Yes	No	Yes	Yes	Yes	Yes	Yes	Yes	No	No
	Needs central authentication	Yes	No	Yes	Yes	Yes	No	No	No	No	No
	Needs DHCP server	Yes	No	Yes	Yes	Yes	No	No	No	No	No
IP	Device IP hopping	Yes	No	Yes	Yes	Yes	Yes	Yes	Yes	No	Yes
	Transmitted IP over air *	DIP	DIP	DIP	DIP	DIP	DIP	DIP	DIP	VIP	VIP
	Virtual IP hopping	No	No	No	No	No	No	No	No	Yes	Yes
	IP synchronization	No	No	No	No	No	Yes	Yes	Yes	No	No
	Hopping by time	No	Yes	No	No	No	Yes	Yes	Yes	Yes	Yes
	Hopping by event	No	No	Yes	Yes	Yes	No	No	No	No	Yes
	Hopping range	Low	High	Low	Low	Low	High	Low	High	Low	High

* DIP stands for device IP and VIP for virtual IP.

**Table 2:** The overhead (time) of generating a sha-256 based synchronous signature.

	Time Spent (μs)
Hash AND Update IP	10.5
Generate a new hash parameter	0.1

**Table 3:** Comparison of our algorithm and other IP hopping related methods.

Method	Infrastructure Support	Wiretapping Capability	Scanning Attack	MIMT Attack	Additional Packets	Hopping on Time	Hopping on Event	Hopping Range
Our Method	x	√	√	√	x	√	√	High
Kravtsov [19]	√	√	√	√	x	x	x	Low
Chang [29]	√	√	√	√	x	x	x	Low
Zhao [12]	√	x	√	x	x	x	x	Low
Albanese [23]	√	√	√	√	x	√	x	Low
Yun He [20]	√	√	√	√	x	x	√	Low
Park [21]	√	x	x	x	x	√	x	High
Sun [15]	√	√	√	√	√	x	√	Low

## Data Availability

Not applicable.

## References

[1] Kanellopoulos A., Vamvoudakis K.G. (2020). A Moving Target Defense Control Framework for Cyber-Physical Systems. IEEE Trans. Autom. Control.

[2] Sengupta S., Chowdhary A., Sabur A., Alshamrani A., Huang D., Kambhampati S. (2020). A Survey of Moving Target Defenses for Network Security. IEEE Commun. Surv. Tutor..

[3] Navas R.E., Cuppens F., Cuppens N.B., Toutain L., Papadopoulos G.Z. (2020). MTD, Where Art Thou? A Systematic Review of Moving Target Defense Techniques for IoT. IEEE Internet Things J..

[4] Fu Y., Ding Z. Hybrid channel access with CSMA/CA and SOTDMA to improve the performance of MANET. Proceedings of the IEEE 17th International Conference on Communication Technology (ICCT).

[5] Fu Y., Ding Z., Wang D. A new type of portable MANET terminal with two modes of CSMA and SOTDMA. Proceedings of the 3rd IEEE International Conference on Computer and Communications (ICCC).

[6] Cho J., Sharma D.P. (2020). Toward Proactive, Adaptive Defense: A Survey on Moving Target Defense. IEEE Commun. Surv. Tutor..

[7] Mohsin M., Prakash R. IP address assignment in a mobile ad hoc network. Proceedings of the MILCOM 2002.

[8] Tang H., Sun Q.T., Yang X., Long K. (2018). A Network Coding and DES Based Dynamic Encryption Scheme for Moving Target Defense. IEEE Access.

[9] Clark A., Sun K., Poovendran R. Effectiveness of IP address randomization in decoy-based moving target defense. Proceedings of the 52nd IEEE Conference on Decision and Control.

[10] Azab M., Hassan R., Eltoweissy M. ChameleonSoft: A moving target defense system. Proceedings of the 7th International Conference on Collaborative Computing: Networking, Applications and Worksharing (CollaborateCom).

[11] Larsen P., Homescu A., Brunthaler S., Franz M. SoK: Automated Software Diversity. Proceedings of the IEEE Symposium on Security and Privacy.

[12] Taguinod M., Doupé A., Zhao Z., Ahn G. Toward a Moving Target Defense for Web Applications. Proceedings of the IEEE International Conference on Information Reuse and Integration.

[13] Porter J., Albassam E.A. Decentralized Approach to Architecture-Based Self-Protecting Software Systems. Proceedings of the 10th Annual Computing and Communication Workshop and Conference (CCWC).

[14] Li Y., Dai R., Zhang J. Morphing communications of Cyber-Physical Systems towards moving-target defense. Proceedings of the IEEE International Conference on Communications (ICC).

[15] Sun J., Sun K. DESIR: Decoy-enhanced seamless IP randomization. IEEE INFOCOM 2016. Proceedings of the 35th Annual IEEE International Conference on Computer Communications.

[16] Dunlop M., Groat S., Urbanski W., Marchany R., Tront J. MT6D: A Moving Target IPv6 Defense. Proceedings of the MILCOM 2011 Military Communications Conference.

[17] Antonatos S., Akritidis P., Markatos E.P., Anagnostakis K.G. (2007). Defending against hitlist worms using network address space randomization. Comput. Netw..

[18] Jafarian J.H., Al-Shaer E., Duan Q. Adversary-aware IP address randomization for proactive agility against sophisticated attackers. Proceedings of the IEEE Conference on Computer Communications (INFOCOM).

[19] Krylov V., Kravtsov K., Sokolova E., Lyakhmanov D. SDI defense against DDoS attacks based on IP Fast Hopping method. Proceedings of the International Science and Technology Conference (Modern Networking Technologies) (MoNeTeC).

[20] He Y., Zhang M., Yang X., Sun Q.T., Luo J., Yu Y. (2020). The Intelligent Offense and Defense Mechanism of Internet of Vehicles Based on the Differential Game-IP Hopping. IEEE Access.

[21] Chang S., Park Y., Babu B.B.A. (2019). Fast IP Hopping Randomization to Secure Hop-by-Hop Access in SDN. IEEE Trans. Netw. Serv. Manag..

[22] Shaer E., Duan Q., Jafarian J.H. (2012). Random host mutation for moving target defense. Security and Privacy in Communication Networks.

[23] Albanese M., De Benedictis A., Jajodia S. A moving target defense mechanism for MANETs based on identity virtualization. Proceedings of the IEEE Conference on Communications and Network Security (CNS).

[24] Floissac N., L’Hyver Y. From AES-128 to AES-192 and AES-256, How to Adapt Differential Fault Analysis Attacks on Key Expansion. Proceedings of the Workshop on Fault Diagnosis and Tolerance in Cryptography.

[25] Shivkumar S., Umamaheswari G. Performance Comparison of Advanced Encryption Standard (AES) and AES Key Dependent S-Box. Proceedings of the Simulation Using MATLAB, International Conference on Process Automation, Control and Computing.

[26] Yu L., Zhang D., Wu L., Xie S., Su D., Wang X. AES Design Improvements Towards Information Security Considering Scan Attack. Proceedings of the 2018 17th IEEE International Conference on Trust, Security and Privacy in Computing and Communications/12th IEEE International Conference on Big Data Science And Engineering (TrustCom/BigDataSE).

[27] Hajihassani O., Monfared S.K., Khasteh S.H., Gorgin S. (2019). Fast AES Implementation: A High-Throughput Bitsliced Approach. IEEE Trans. Parallel Distrib. Syst..

[28] Tamang J. (2021). Dynamical Properties of Ion-Acoustic Waves in Space Plasma and Its Application to Image Encryption. IEEE Access.

[29] García-Guerrero E.E., Inzunza-González E., López-Bonilla O.R., Cárdenas-Valdez J.R., Tlelo-Cuautle E. (2020). Randomness improvement of chaotic maps for image encryption in a wireless communication scheme using PIC-microcontroller via Zigbee channels. Chaos Solitons Fractals.

[30] Rathor M., Sengupta A. (2020). IP Core Steganography Using Switch Based Key-Driven Hash-Chaining and Encoding for Securing DSP Kernels Used in CE Systems. IEEE Trans. Consum. Electron..

[31] Erdem E., Sandıkkaya M.T. (2019). OTPaaS—One Time Password as a Service. IEEE Trans. Inf. Forensics Secur..

[32] Wu L., Cai H.J., Li H. (2021). SGX-UAM: A Secure Unified Access Management Scheme with One Time Passwords via Intel SGX. IEEE Access.

[33] Maidhili R., Karthik G. Energy Efficient and Secure Multi-User Broadcast Authentication Scheme in Wireless Sensor Networks. Proceedings of the International Conference on Computer Communication and Informatics (ICCCI).

[34] Kwon T., Hong J. (2010). Secure and Efficient Broadcast Authentication in Wireless Sensor Networks. IEEE Trans. Comput..

[35] Najib A.F., Rachmawanto E.H., Sari C.A., Sarker K., Rijati N. A Comparative Study MD5 and SHA1 Algorithms to Encrypt REST API Authentication on Mobile-based Application. Proceedings of the International Conference on Information and Communications Technology (ICOIACT).

